# Public knowledge about dementia risk reduction in Norway

**DOI:** 10.1186/s12889-022-14433-w

**Published:** 2022-11-08

**Authors:** Grete Kjelvik, Anne Marie Mork Rokstad, Josephine Stuebs, Pernille Thingstad, Kay Deckers, Sebastian Köhler, Geir Selbæk

**Affiliations:** 1 Norwegian National Centre for Ageing and Health, Vestfold Hospital Trust, PO Box 2136, Tønsberg, 3103 Norway; 2 Faculty of Health Sciences and Social Care, Molde University College, Molde, Norway; 3 Institute of Clinical Medicine, University of Oslo, Oslo, Norway; 4 Department of Neuroscience and Movement Science (INB), Faculty of Medicine and Health Sciences, Norwegian University of Science and Technology, Trondheim, Norway; 5 Department of Health and Welfare, Trondheim Municipality, Trondheim, Norway; 6 Department of Psychiatry and Neuropsychology, School for Mental Health and Neuroscience, Alzheimer Centrum Limburg, Maastricht University, Maastricht, The Netherlands; 7 Department of Geriatric Medicine, Oslo University Hospital, Oslo, Norway

**Keywords:** Prevention, Dementia, Risk factors, Lifestyle, Public knowledge, Awareness, Brain health

## Abstract

**Background:**

Several modifiable lifestyle risk factors for dementia have been identified, but it is unclear how much the Norwegian public knows about the relationship between lifestyle and brain health. Therefore, this study aimed to investigate knowledge about modifiable dementia risk and protective factors and beliefs and attitudes towards dementia and dementia risk reduction in a randomly selected subsample of the Norwegian population.

**Methods:**

The total sample (*n* = 1435) included individuals aged 40–70 years from four counties (Oslo, Innlandet, Nordland and Trøndelag) in Norway. Two online questionnaires were used to measure (1) awareness about dementia risk reduction and (2) an individual`s motivation to change behaviour for dementia risk reduction (MOCHAD-10).

**Results:**

Of the participants, 70% were aware of the potential of dementia risk reduction in general. Physical inactivity (86%), cognitive inactivity (84%) and social isolation (80%) were the most frequently recognised dementia risk factors. On the other hand, diabetes (26%), coronary heart disease (19%), hearing loss (18%) and chronic kidney disease (7%) were less often recognised as dementia risk factors. Comparing men and women, the only significant difference was that women were more likely to report parents with dementia as a risk factor compared to men. Gender, age and educational differences were seen in beliefs and attitudes towards dementia prevention:women reported more negative feelings and attitudes towards dementia than men;those aged 40–49 years – more likely than older age groups – reported that ‘knowing family members with dementia’ or ‘having risk factors’ made them believe they had to change their lifestyle and behaviour.

**Conclusions:**

The results indicate that 70% of the Norwegian public are aware of the potential for dementia risk reduction in general. However, there are major gaps in existing knowledge, particularly for cardiovascular risk factors such as hypertension, coronary heart disease, hypercholesterolemia and metabolic factors (diabetes, obesity). These findings underline the importance of further informing the Norwegian public about lifestyle-related risk and protective factors of dementia. Differences in beliefs and attitudes towards dementia risk prevention by age, gender and education require tailored public risk reduction interventions.

**Supplementary Information:**

The online version contains supplementary material available at 10.1186/s12889-022-14433-w.

## Introduction

In Norway, the number of persons with dementia was estimated to be 101,118 in 2020, and that number is expected to more than double by 2050 [1]. Dementia is one of the key health care challenges in society today, both nationally and internationally. Since there is no curative treatment available for dementia, alternative ways of decreasing the individual and societal impact of dementia disorders are sought. Next to non-modifiable risk factors such as age, sex and genetics, there is evidence that modifiable risk factors contribute to dementia risk. A recent report estimated that up to 40% of dementia cases could potentially be delayed or prevented by interventions directed at the most common risk factors such as midlife obesity, midlife hypertension, diabetes, depression, smoking, physical inactivity and social isolation [2]. Combined with less education in early life, these factors have consistently been found to increase dementia risk in observational studies and meta-analyses [2, 3]*.*

It is well-established that individuals can make important contributions to improving their present health or decreasing the risk of future health problems [4, 5]*.* However, this might be particularly challenging in the context of dementia, where risk accumulates throughout one’s lifetime, and health behaviours may need to be modified 30 or 40 years prior to the onset of the disease. Four reviews suggest that knowledge about the potential for dementia risk reduction is poor in the general population [6–9]. The review by Cations and others in 2018 included 33 studies from Europe, the US, Eastern Asia, Israel and Australia, and half of the respondents reported that dementia is a normal part of ageing and not preventable [7]. However, cognitive leisure activities, in particular, appeared to be understood as a good candidate for dementia prevention [7]*.* The WHO guidelines call for action towards dementia risk reduction [10], but so far, only a few countries have conducted public health awareness campaigns focused on dementia risk reduction as an important first step towards behavioural changes [11, 12], and most participants (> 70%) wanted information on improving their brain health [11]*.*

A survey investigating public knowledge about dementia risk reduction has never been conducted in Norway. Norwegian national lifestyle intervention strategies to prevent or postpone dementia must be acceptable to the public in Norway. To create such strategies it is vital to determine the level of knowledge about dementia risk prevention, beliefs and attitudes towards dementia and dementia risk reduction. Therefore, the present study aims to investigate knowledge about dementia risk reduction, beliefs and attitudes towards dementia and dementia risk reduction in a randomly selected subsample of the Norwegian population.

## Methods

### Study design and recruitment

The present study has a cross-sectional design using a public survey to collect data. A total of 7738 individuals were randomly selected from the Norwegian population register and invited to fill out an online questionnaire. The total sample included individuals aged 40–70 years from the counties of Oslo, Innlandet, Nordland and Trøndelag. The counties were selected to represent urban and rural areas, large cities and small municipalities, inland and coastal regions.

All participants received a letter with an invitation to fill out the questionnaire (www.hjernehelse.info/skjema). Data collection was performed on the Online Service for Sensitive Data (TSD) facilities, owned by the University of Oslo and operated and developed by the TSD service group at the University of Oslo, IT Department (USIT). The TSD is designed for storing and post-processing sensitive data in compliance with the Norwegian Personal Data Act and Health Research Act. We used a version of the UiO web questionnaire that interfaces with the governmental ID portal for login, allowing secure data harvesting and strong identification of the respondents. The system automatically registered the response time for filling in the questionnaire for each participant. Information about the project was published at the project’s homepage (www.hjernehelse.info). Survey reminders were sent by SMS to individuals who had not reserved themselves against advertisements (*n* = 5360 persons). Each participant could answer the questionnaire only once.

### Measurements

Participants were asked to answer questions regarding their gender, age, marital status and level of education as part of the survey. Level of education was obtained by self-assessment of the highest finalized degree and categorized into five degrees (Table 1). Two questionnaires were used to collect data on the public knowledge and level of awareness of dementia risk and the beliefs and attitudes towards dementia: one questionnaire to measure the awareness of dementia risk reduction [13] and the Motivation to Change Behaviour for Dementia Risk Reduction *(MOCHAD-10)* [14]*.* The awareness questionnaire assesses knowledge of 14 risk and protective factors. The 12 modifiable risk and protective factors from the Lifestyle for BRAin Health (LIBRA) score are included [11]. Additionally, we included two custom-made items on social isolation and hearing loss as potential dementia risk factors (Additional file 1) [15]. The participants were also asked a general question about their knowledge of dementia. The Motivation to Change Lifestyle and Health Behaviours for Dementia risk reduction (MCLHB-DRR) scale measures the beliefs and attitudes towards dementia and dementia risk reduction [16]. The MCLHB-DRR scale has been cross-culturally validated in other countries such as Turkey [17] and The Netherlands [18]. In the United Kingdom, a short, reliable and robust two-factor, 10-item scale, Motivation to Change Behaviour for Dementia Risk Reduction (MOCHAD-10), was developed and chosen for use in our study [14] (Additional file 2). The 10-item scale represents two concepts of positive and negative elements related to motivation to change lifestyle, covering a range of beliefs and feelings. Factor 1 = Positive cues to action (questions 1–5) and factor 2 = Negative cues to action (questions 6–10) (Additional file 2). For both questionnaires, participants were asked to what extent they agreed or disagreed on a five-point Likert scale ranging from “agree strongly”, “agree”, “Neither agree nor disagree”, “disagree” to “disagree strongly”. The participants were informed that there were no right or wrong answers to any questions and were encouraged to answer them as honestly and openly as possible.

**Table 1:** Characteristics of the total sample

**Sample characteristics**	**Total sample** ***N* = 1435**
*Age group (year), n (%)*	
40–49	367 (25.6%)
50–59	510 (35.5%)
60–70	559 (38.9%)
Gender, men/women (%)	617/818 (43 /57)
*Educational level, n (%)*	
9–10 years of compulsory primary and lower secondary school	41 (2.8%)
1 or 2 years of academic or vocational school	106 (7.3%)
3 years of academic or vocational school	143 (9.9%)
3–4 years vocational school/apprentice (upper secondary/sixth form college)	208 (14.4%)
College or university, less than four years	381 (26.3%)
College or university, four years or more	556 (38.4%)
*Marital status, n (%)*	
Married or living with a significant other	1104 (76.3%)
Not or never been married	139 (9.6%)
Divorced	157 (10.9%)
Widowed	33 (2.3%)

Both questionnaires underwent the same translation procedure. They were translated into Norwegian by two psychiatrists and a nurse. The three translations of each questionnaire were collated by a fourth person (the project leader) into the final Norwegian versions, which were translated back into English by a native English speaker. These versions were compared with the original English versions in a consensus meeting with the translators, and they were found to be comparable. The final Norwegian versions were used in this study*.*

### Statistical analysis

Chi-square tests were used to examine whether the demographic variables gender, age groups (40–49 years, 50–59 years and 60–70 years) and educational level (lower education level = 9 to 13 years and higher education level = 13 + years) were associated with knowledge of risk and protective factors, level of awareness, and beliefs and attitudes towards dementia and dementia risk reduction. Comparison of the level of general knowledge of dementia with knowledge of risk and protective factors was done using Chi-square tests. All analyses were performed in IBM SPSS Statistics Version 27 (IBM SPSS; www.spss.com), and the level of statistical significance used was *p* < 0.05, in two-tailed tests.

For the general question about dementia risk reduction, answers to the question "There is nothing anyone can do to reduce their risks of getting dementia" were coded as unaware (“agree strongly”, “agree”, “neither agree nor disagree”) and aware (“disagree”, “disagree strongly”).

Correct recognition of the presented risk and protective factors includes “strongly agree” and “agree”. Incorrect recognition of the presented risk and protective factors includes both categories “strongly disagree”, “disagree” and “neither agree/nor disagree”.

## Results

### Demographics

Of the 7738 invited, a total of 1435 (18.5%) subjects participated in the study by completing the questionnaire. No significant differences were observed in age and gender between the participating public and the non-participating public (*n* = 6302). Of the total sample (*n* = 1435), 57% were women, 25.6% were in the age group 40–49 years, 35.5% were 50–59 years, and 38.9% were in the age group 60–70 years. For characteristics of the total sample, see Table 1. The mean age and propotion of women in the group 40–70 years in the selected counties compared with the entire country were 54.2 years and 54.2 years, and 49.2% vs. 49.1%, respectively. Flowcharts of the invited and participating samples are presented in Additional file 3. Mean response time for filling in the questionnaire for the respondents was eight minutes (SD 6.4). A total of 80% of participants reported that they presently or previously had family members and/or friends with dementia. Most respondents (76%) in our study indicated that they would welcome more information on improving their brain health.

### General awareness and dementia knowledge

Of the total sample, 70% of the respondents (*n* = 1297) were aware of the potential for dementia risk reduction in general. In addition, the participants were asked a general question about their knowledge about dementia. Four percent of the participants reported that they knew a great deal about dementia, 39% reported that they knew some, and 53% reported that they knew nothing about dementia. Furthermore, higher awareness of dementia risk reduction was significantly more often recognised among the participants with good knowledge than participants with no knowledge (*p* < 0.05). In addition, women were significantly more aware of dementia risk reduction than men (*p* = 0.02).

### Knowledge of dementia risk and protective factors

**Fig. 1:**
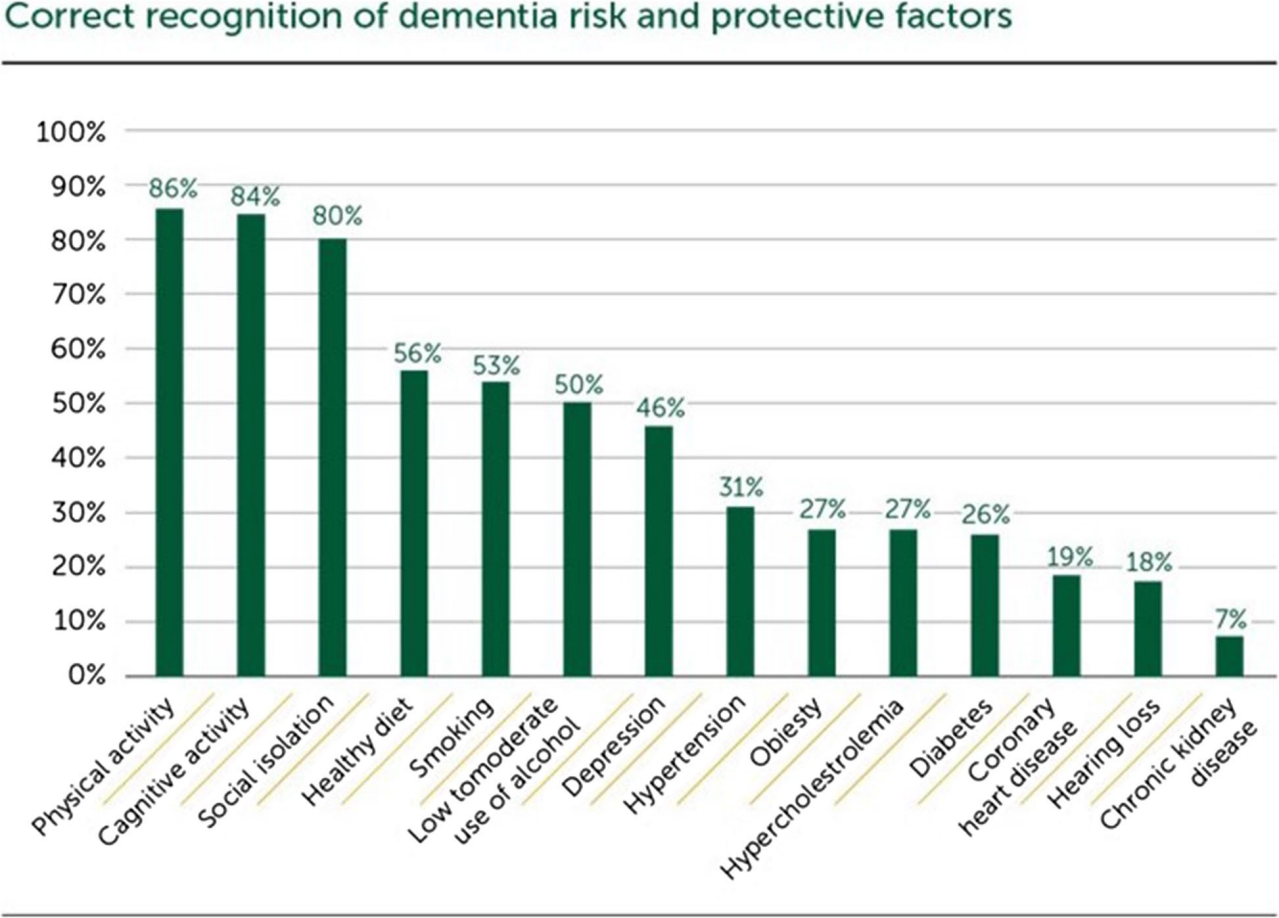
Correct recognition of dementia risk factors and protective factors for the total sample (*n* = 1435)

Half of the sample (50%) identified zero to six of the 15 factors, and only 1.5% identified all factors correctly. Physical inactivity (86%), cognitive inactivity (84%) and social isolation (80%) were the most frequently recognised dementia risk factors. On the other hand, diabetes (26%), coronary heart disease (19%), hearing loss (18%) and chronic kidney disease (7%) were less often recognised as dementia risk factors. All the presented dementia risk and protective factors were significantly more often recognised among the participants with good dementia knowledge (“great” and “know some”) than participants with no knowledge (“no knowledge” and “don’t know”) (*p* < 0.05). See Fig. 1 for correct recognition of presented risk factors and protective factors for dementia by responding “strongly agree” or “agree” to the question.

### Gender, age and education

**Fig. 2:**
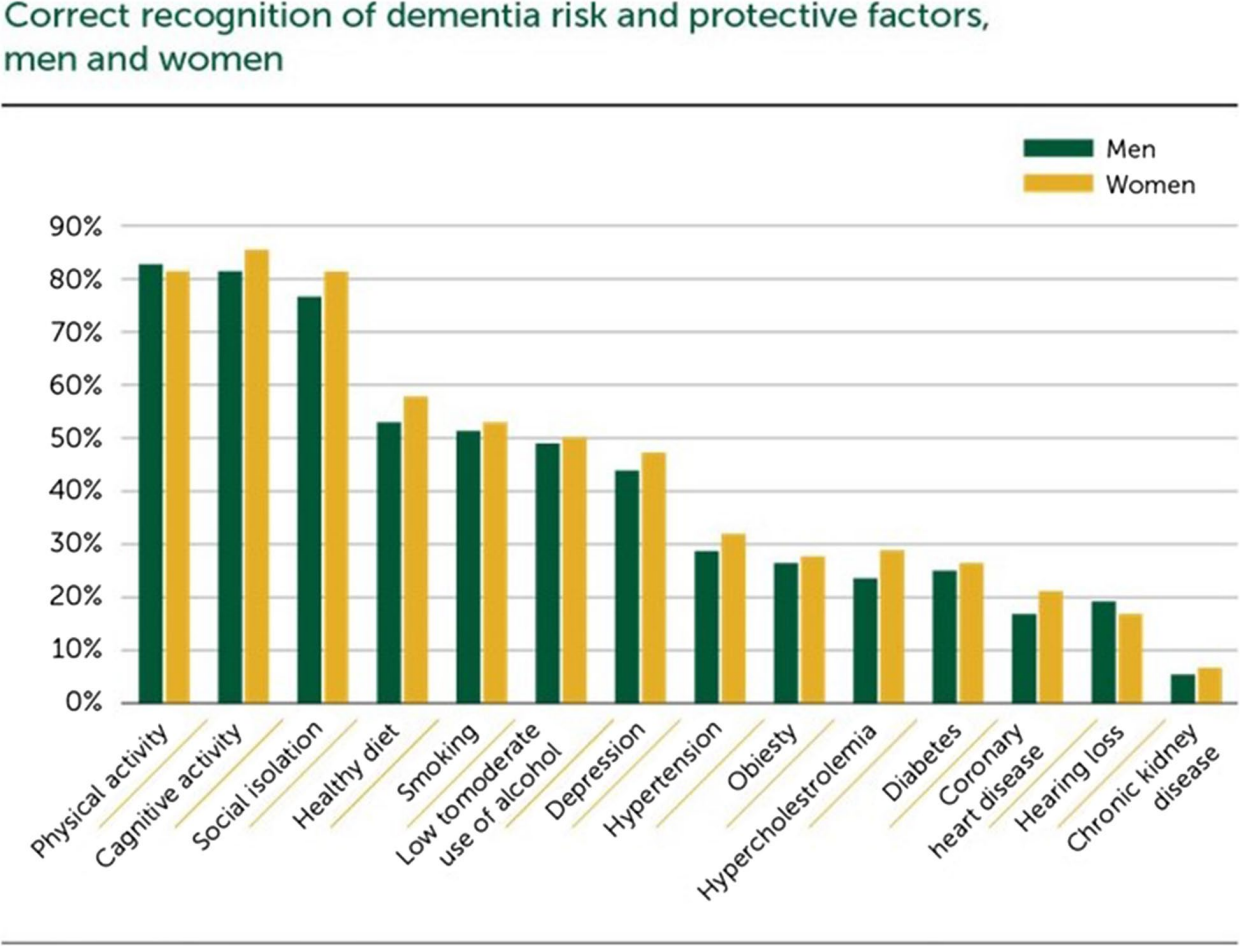
Identified risk and protective factors for men and women separately

A comparison of men and women showed that women more frequently identified parents with dementia (61.8% versus 38.2%, *p* < 0.0005), low cognitive activity (58% versus 42%, *p* = 0.035), coronary heart disease (62.4% versus 37.6%, *p* = 0.043), hypercholesterolemia (61.4% versus 38.6%, *p* = 0.043) and social isolation (58.6% versus 41.4%, *p* = 0.015) as risk factors for dementia (see Fig. 2).

Individuals in the youngest age group (40–49 years, *n* = 367, 77.8%) were significantly more likely than individuals in the middle age group and the oldest age group (*n* = 559, 67.3%) to report having a parent with dementia as a risk factor (*p* = 0.028). Furthermore, individuals in the oldest group were significantly more likely than individuals in the middle-aged group to recognise smoking (*p* = 0.001), hypercholesterolemia (*p* < 0.005) and hypertension (*p* = 0.003) as risk factors for dementia. No other significant differences between age groups in the perception of risk factors were identified.

According to level of education, all the risk factors except hearing loss were less often reported by the participants with lower education (9 to 13 years) than the participants with higher education (13 + years) (*p* < 0.05).

### Beliefs and attitudes towards dementia and dementia risk reduction

**Fig. 3:**
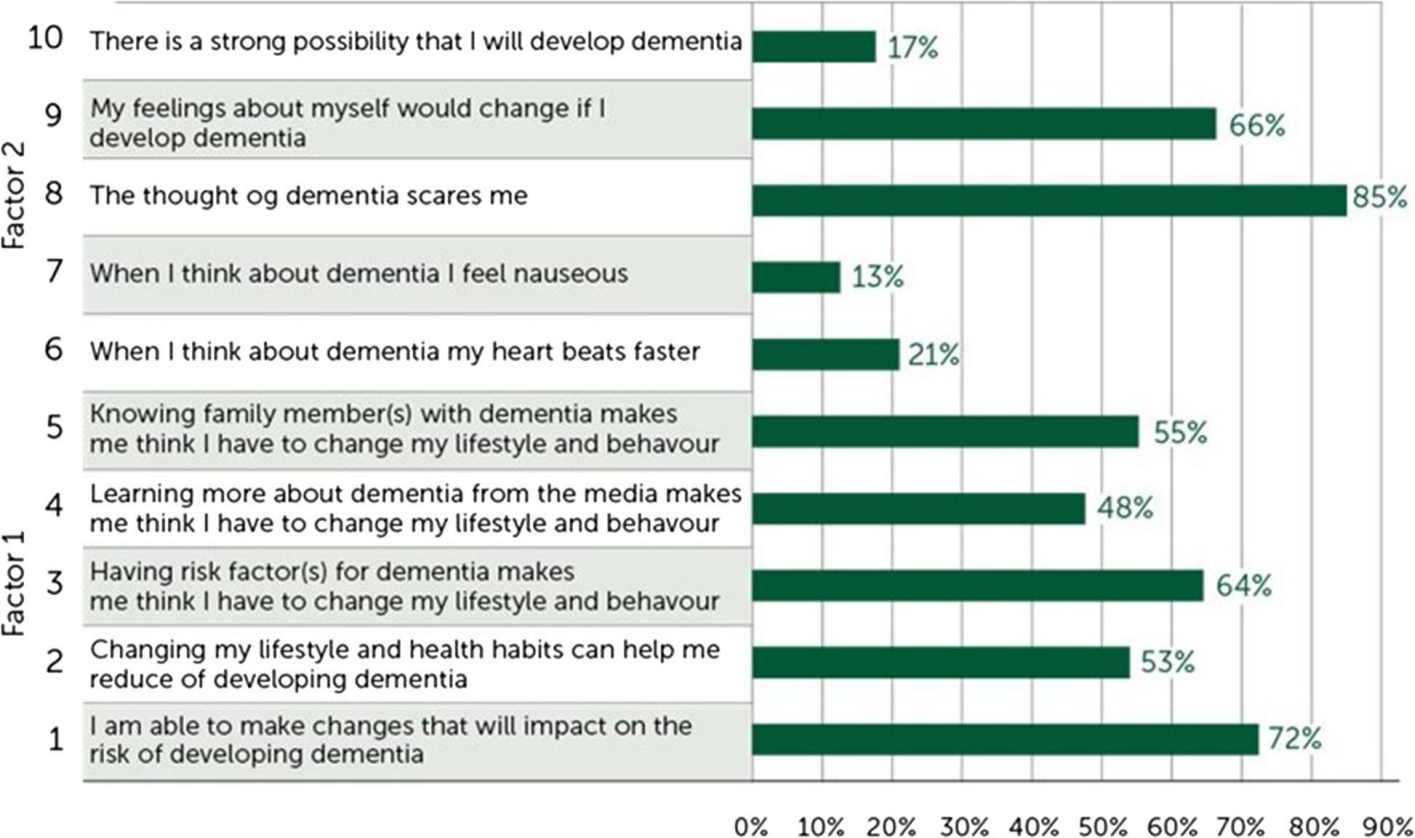
Beliefs and attitudes towards dementia and dementia risk reduction. Positive cues to action (items 1–5) and negative cues to action (items 6–10)

A total of 72% of the participants (*n* = 1430) answering the MOCHAD-10 questionnaire strongly believed they were able to change their lifestyle to reduce the risk of developing dementia, while most respondents (85%) reported that the thought of dementia scared them (Fig. 3).

### Gender, age and education

A comparison of the responses of men and women showed that women were significantly more likely than men to endorse any negative cue to action (questions 6–10), as well as two of the positive cues to action “Knowing family member(s) with dementia makes me think I have to change my lifestyle and behaviour”(*p* = 0.02), “I am able to make differences that will change the risk of developing dementia” (*p* = 0.05) to dementia prevention.

People aged 40–49 years were significantly more likely than older age groups to endorse the two positive cues to action (“Knowing family member(s) with dementia makes me think I have to change my lifestyle and behaviour” [*p* < 0.05] and “Having risk factors for dementia makes me think I have to change my lifestyle and behaviour” [*p* = 0.01]). In contrast, the oldest age group (60–70 years) less often reported that there was a strong possibility that they would develop dementia compared to younger age groups (*p* = 0.009).

Those highly educated (13 years +) were significantly more likely than those with lower education (9 to 13 years) to endorse two positive cues to action (“I am able to make differences that will change the risk of developing dementia” [*p* < 0.005], “Knowing family member(s) with dementia makes me think I have to change my lifestyle and behaviour” [*p* = 0.003]) and two negative cues to action (“When I think about dementia, my heart beats faster”[*p* = 0.002], “My feelings about myself would change if I develop dementia”[*p* = 0.02]).

## Discussion

This study assessed knowledge about dementia risk reduction and beliefs and attitudes towards dementia and dementia prevention in a Norwegian population. The results showed that the level of general knowledge about dementia was moderate in the public. However, the majority (70%) of participants were aware of the potential of dementia risk reduction in general, with differences in knowledge of risk and protective factors and attitudes by age, sex and educational level.

The level of awareness is essential regarding behavioural changes and awareness strategies for dementia prevention. Our findings indicate a high level of awareness in Norway, which is higher than a similar study of the Flemish (35%) and Dutch (44%) populations [11, 13, 19] and the international review by Cations [7]. For several decades, Norwegian central health authorities have had dementia plans with strategies for meeting the future challenges of dementia. Since public awareness has been addressed as part of these plans, it might have affected the level of awareness of dementia prevention in Norway [20].

The protective factors of physical activity and cognitive activity were among those most commonly recognised, as seen in other studies [5, 7, 11]. However, there were large gaps in knowledge about other risk and protective factors of dementia. Major gaps in knowledge existed, particularly for factors such as hearing loss, metabolic factors (diabetes, obesity), chronic kidney disease and cardiovascular disease (hypertension, coronary heart disease, hypercholesterolemia). A review of population-based surveys in Europe, Australia, the US, Eastern Asia and Israel also reported the lack of knowledge about cardiovascular risk factors [7], and recognition of cardiovascular risk factors for dementia is generally very limited [7, 8, 11].

People aged 40–49 years significantly more often than older people reported family history as the major risk factor for dementia. They also believed that having a family history of dementia meant they had to change their lifestyle and behaviour for dementia risk reduction more often than older people. Family history and genetics are non-modifiable risk factors but might be a motivational factor for lifestyle changes to reduce dementia risk. Among the higher-educated participants, significantly more risk and protective factors were identified in general, except the knowledge about hearing. General awareness of dementia risk reduction was also significantly more common among higher-educated participants. Differences in knowledge of dementia risk prevention in relation to education and age are also seen in other studies [11], indicating that campaigns need to be customized related to age and education/socioeconomic status. Epidemiological research indicates that the risk for dementia shows inequality, as people with low socioeconomic status, immigrants and people with non-Western ethnic backgrounds have a higher risk of dementia [21], which might be partially explained by their having a poorer lifestyle [22].

Both internal and external factors might drive motivation to change lifestyle. The results indicated a general pattern with more negative feelings and attitudes towards dementia among women than men. However, 85% of all participants reported that dementia scares them. Furthermore, 72% of the participants strongly believed that they were able to change their lifestyle to reduce the risk of developing dementia, representing positive beliefs and attitudes towards dementia risk reduction. Large individual differences in attitudes and beliefs in dementia prevention indicate the need for more in-depth information at individual levels. Therefore, qualitative studies with interviews and focus groups should be recommended to learn about individual barriers, facilitators and preferences for dementia risk reduction.

In its global action plan on the public response to dementia, the World Health Organization recommends raising awareness about the modifiable risk factors of dementia [10]. Most respondents (76%) in our study indicated that they would welcome more information on improving their brain health. Successful campaigns or interventions in Norway need to be accompanied by approaches to overcome individual differences in motivation level by age and socioeconomic status. It is also important to choose a positive approach, for example, by using words such as “brain health” for a greater probability of raising awareness of dementia risk reduction among the people in midlife. On the other hand, the scary thought of dementia was the strongest positive cue and might be a strong motivational force for lifestyle changes at an individual level. Fear might promote better health behaviours, but on the other hand defensive reactions might also be linked to less intentions to change behaviour [23]. The major differences between the target groups in motivational factors must also be taken into account when planning upcoming campaigns. Filling knowledge gaps will be crucial to moving forward in dementia prevention in the general public, and appropriate information that vascular health behaviours may reduce risk especially needs to be effectively communicated in Norway.

However, evaluation studies of public health campaigns indicate that groups with a higher risk of dementia such as immigrants with non-Western-ethnic background or people living in poverty are harder to reach [19]. As the participants in the current study are mainly highly educated people, the results from the survey must be interpreted with caution as a knowledge base for information campaigns. The challenge is to design dementia risk reduction campaigns tailored to people in higher risk groups who are also in the harder-to-reach groups, as their health literacy is more limited.

The study has several strengths and weaknesses. The response rate in the study was relatively low (18.5%), although this is not unusual for survey studies. Additionally, the sample seems biased towards the most motivated people based on high education, and many of them have a family member with dementia, limiting generalizability. However, we studied variation in awareness and knowledge of these characteristics to learn about such differences and make recommendations for future campaigns. Some strengths of this study are the random and anonymous sample and the standardized questionnaires used that make the results easily comparable with studies from other countries.

## Conclusion

The present study indicates that the majority of the Norwegian public is aware of the relation between lifestyle and brain health. However, major gaps in knowledge of lifestyle-related risk factors existed, particularly for factors such as hearing loss, diabetes, obesity and cardiovascular disease (hypertension, coronary heart disease, hypercholesterolemia). The findings underline the importance of further informing the Norwegian public about lifestyle-related risk factors and the prevention of dementia. Campaigns need to be customized according to age and education/socioeconomic status.

## Supplementary Information

**Additional file 1. **Dementia awareness questionnaire.

**Additional file 2. **Motivationto Change Lifestyle for Dementia Risk Reduction (MOHAD-10).

**Additional file 3. **Flowchart of the invited and participating sample.

## Data Availability

The dataset used and/or analyzed during the current study are available from the corresponding author on reasonable request.

## References

[1] Gjora L, Strand BH, Bergh S, Borza T, Braekhus A, Engedal K (2021). Current and Future Prevalence Estimates of Mild Cognitive Impairment, Dementia, and Its Subtypes in a Population-Based Sample of People 70 Years and Older in Norway: The HUNT Study. J Alzheimers Dis.

[2] Livingston G, Huntley J, Sommerlad A, Ames D, Ballard C, Banerjee S (2020). Dementia prevention, intervention, and care: 2020 report of the Lancet Commission. Lancet.

[3] Vos SJB, van Boxtel MPJ, Schiepers OJG, Deckers K, de Vugt M, Carriere I (2017). Modifiable Risk Factors for Prevention of Dementia in Midlife, Late Life and the Oldest-Old: Validation of the LIBRA Index. J Alzheimers Dis.

[4] Grande G, Qiu C, Fratiglioni L (2020). Prevention of dementia in an ageing world: Evidence and biological rationale. Ageing Res Rev.

[5] Smith BJ, Ali S, Quach H (2014). Public knowledge and beliefs about dementia risk reduction: a national survey of Australians. BMC Public Health.

[6] Norton S, Matthews FE, Barnes DE, Yaffe K, Brayne C (2014). Potential for primary prevention of Alzheimer's disease: an analysis of population-based data. Lancet Neurol.

[7] Cations M, Radisic G, Crotty M, Laver KE (2018). What does the general public understand about prevention and treatment of dementia? A systematic review of population-based surveys. PLoS One.

[8] Parial LL, Lam SC, Ho JYS, Suen LKP, Leung AYM (2021). Public knowledge of the influence of modifiable cardiovascular risk factors on dementia: a systematic literature review and meta-analysis. Aging Ment Health.

[9] Cahill S, Pierce M, Werner P, Darley A, Bobersky A (2015). A systematic review of the public's knowledge and understanding of Alzheimer's disease and dementia. Alzheimer Dis Assoc Disord.

[10] WHO WHO (2019). Risk Reduction of Cognitive Decline and Dementia.

[11] Heger I, Deckers K, van Boxtel M, de Vugt M, Hajema K, Verhey F (2019). Dementia awareness and risk perception in middle-aged and older individuals: baseline results of the MijnBreincoach survey on the association between lifestyle and brain health. BMC Public Health.

[12] Van Asbroeck S, van Boxtel MPJ, Steyaert J, Kohler S, Heger I, de Vugt M (2021). Increasing knowledge on dementia risk reduction in the general population: results of a public awareness campaign. Prev Med.

[13] Heger I, Kohler S, van Boxtel M, de Vugt M, Hajema K, Verhey F (2020). Raising awareness for dementia risk reduction through a public health campaign: a pre-post study. BMJ Open.

[14] Oliveira D, Aubeeluck A, Stupple E, Kim S, Orrell M (2019). Factor and reliability analysis of a brief scale to measure motivation to change lifestyle for dementia risk reduction in the UK: the MOCHAD-10. Health Qual Life Outcomes.

[15] Livingston G, Sommerlad A, Orgeta V, Costafreda SG, Huntley J, Ames D (2017). Dementia prevention, intervention, and care. Lancet.

[16] Kim S, Sargent-Cox K, Cherbuin N, Anstey KJ (2014). Development of the motivation to change lifestyle and health behaviours for dementia risk reduction scale. Dement Geriatr Cogn Dis Extra.

[17] Zehirlioglu L, Erunal M, Akyol MA, Mert H, Hatipoglu NS, Kucukguclu O (2019). Turkish Version of the Motivation for Changing Lifestyle and Health Behavior for Reducing the Risk of Dementia Scale. J Neurosci Nurs.

[18] Joxhorst T, Vrijsen J, Niebuur J, Smidt N (2020). Correction to: Cross-cultural validation of the motivation to change lifestyle and health behaviours for dementia risk reduction scale in the Dutch general population. BMC Public Health.

[19] Steyaert J, Deckers K, Smits C, Fox C, Thyrian R, Jeon YH (2021). Putting primary prevention of dementia on everybody's agenda. Aging Ment Health.

[20] Norwegian Ministry of Health and Care Services. Dementia Plan 2025; 2020. Available from: https://www.regjeringen.no/no/dokumenter/demensplan-2025/id2788070/.

[21] Parlevliet JL, Uysal-Bozkir O, Goudsmit M, van Campen JP, Kok RM, Ter Riet G (2016). Prevalence of mild cognitive impairment and dementia in older non-western immigrants in the Netherlands: a cross-sectional study. Int J Geriatr Psychiatry.

[22] Deckers K, Cadar D, van Boxtel MPJ, Verhey FRJ, Steptoe A, Kohler S (2019). Modifiable Risk Factors Explain Socioeconomic Inequalities in Dementia Risk: Evidence from a Population-Based Prospective Cohort Study. J Alzheimers Dis.

[23] Moussaoui LS, Claxton N, Desrichard O (2021). Fear appeals to promote better health behaviors: an investigation of potential mediators. Health Psychol Behav Med.

