# Supplementary material for: Public knowledge about dementia risk reduction in Norway
Source: BMC Public Health. 2022 Nov 8;22:2046. doi: 10.1186/s12889-022-14433-w (PMC9644554; doi:10.1186/s12889-022-14433-w)
Supplement: Supplementary file 1 — Additional file 1. Dementia awareness questionnaire. [file 12889_2022_14433_MOESM1_ESM.docx]

**Additional file 1.** Dementia awareness questionnaire.

Dementia awareness questionnaire (from: Heger I, Deckers K, van Boxtel M, de Vugt M, Hajema K, Verhey F, et al. Dementia awareness and risk perception in middle-aged and older individuals: baseline results of the MijnBreincoach survey on the association between lifestyle and brain health. BMC Public Health. 2019;19(1):678).

--------------------------------------------------------------------------------------------------------------------------

Dementia knowledge

1. Dementia describes a set of symptoms, including loss of memory, sudden mood swings, not remembering who people are, and having trouble finding your words. Alzheimer's disease is one form of dementia. How much would you say you know about dementia?
   - A great deal
   - Quite a lot
   - Some
   - Not very much
   - Nothing at all
   - I don't know
   - I prefer not to answer this question

Dementia risk awareness

*Please state how much you agree or disagree with the following statements.*

1. ‘There is nothing anyone can do to reduce their risks of getting dementia’
   - Agree strongly
   - Agree
   - Neither agree nor disagree
   - Disagree
   - Disagree strongly
2. 'High blood pressure increases your chances of getting dementia'
   - Agree strongly
   - Agree
   - Neither agree nor disagree
   - Disagree
   - Disagree strongly
3. ‘Smoking increases your chances of getting dementia’
   - Agree strongly
   - Agree
   - Neither agree nor disagree
   - Disagree
   - Disagree strongly
4. ‘No or moderate alcohol use lowers your chances of getting dementia’
   - Agree strongly
   - Agree
   - Neither agree nor disagree
   - Disagree
   - Disagree strongly
5. Regular physical activity lowers your chances of getting dementia’
   - Agree strongly
   - Agree
   - Neither agree nor disagree
   - Disagree
   - Disagree strongly
6. ‘Working in a noisy environment increases your chances of getting dementia’
   - Agree strongly
   - Agree
   - Neither agree nor disagree
   - Disagree
   - Disagree strongly
7. ‘Depression increases the chances of getting dementia’
   - Agree strongly
   - Agree
   - Neither agree nor disagree
   - Disagree
   - Disagree strongly
8. ‘Diabetes increases the chances of getting dementia’
   - Agree strongly
   - Agree
   - Neither agree nor disagree
   - Disagree
   - Disagree strongly
9. ‘Being overweight increases the chances of getting dementia’
   - Agree strongly
   - Agree
   - Neither agree nor disagree
   - Disagree
   - Disagree strongly
10. ‘A mentally active lifestyle lowers the chances of getting dementia’
    - Agree strongly
    - Agree
    - Neither agree nor disagree
    - Disagree
    - Disagree strongly
11. ‘Heart disease increases the chances of getting dementia’
    - Agree strongly
    - Agree
    - Neither agree nor disagree
    - Disagree
    - Disagree strongly
12. ‘Kidney disease increases the chances of getting dementia’
    - Agree strongly
    - Agree
    - Neither agree nor disagree
    - Disagree
    - Disagree strongly
13. ‘High cholesterol increases the chances of getting dementia’
    - Agree strongly
    - Agree
    - Neither agree nor disagree
    - Disagree
    - Disagree strongly
14. ‘Healthy diet lowers the chances of getting dementia’
    - Agree strongly
    - Agree
    - Neither agree nor disagree
    - Disagree
    - Disagree strongly
15. `Being alone a lot increases the chances of getting dementia`
    - Agree strongly
    - Agree
    - Neither agree nor disagree
    - Disagree
    - Disagree strongly
