# Supplementary material for: Public knowledge about dementia risk reduction in Norway
Source: BMC Public Health. 2022 Nov 8;22:2046. doi: 10.1186/s12889-022-14433-w (PMC9644554; doi:10.1186/s12889-022-14433-w)
Supplement: Supplementary file 2 — Additional file 2. Motivationto Change Lifestyle for Dementia Risk Reduction (MOHAD-10). [file 12889_2022_14433_MOESM2_ESM.docx]

**Additional file 2. Beliefs and attitudes towards dementia and dementia risk reduction.**

Motivation to Change Lifestyle for Dementia Risk Reduction (MOHAD-10) (from: Oliveira, D., Aubeeluck, A., Stupple, E., Kim, S. and Orrell, M. Factor and reliability analysis of a brief scale to measure motivation to change lifestyle for dementia risk reduction in the UK: the MOCHAD-10. Health Qual Life Outcomes. 2019 May 2; 17(1):75. doi: 10.1186/s12955-019-1143-8).

***Motivation to Change Lifestyle for Dementia Risk Reduction (MOHAD-10)***

Please read the statements below and indicate how much you agree or disagree with them on the five point scale. There are no right or wrong answers to any question as you will be required to rate your own thoughts and beliefs. So please answer them as honestly and openly as possible.

|  | Strongly | Agree | Neither | Disagree | Strongly |
| --- | --- | --- | --- | --- | --- |
|  | Agree |  | Agree |  | Disagree |
|  |  |  | nor |  |  |
|  |  |  | Disagree |  |  |
| 1. I am able to make changes that will impact the risk of developing dementia | ☐ | ☐ | ☐ | ☐ | ☐ |
| 1. Changing my lifestyle and health habits can help me reduce my chance of developing dementia | ☐ | ☐ | ☐ | ☐ | ☐ |
| 1. Having risk factor(s) for dementia makes me think I have to change my lifestyle and behaviour | ☐ | ☐ | ☐ | ☐ | ☐ |
| 1. Learning more about dementia from the media makes me think I have to change my lifestyle and behaviour | ☐ | ☐ | ☐ | ☐ | ☐ |
| 1. Knowing family member(s) with dementia makes me think I have to change my lifestyle and behaviour | ☐ | ☐ | ☐ | ☐ | ☐ |
| 1. When I think about dementia, my heart beats faster | ☐ | ☐ | ☐ | ☐ | ☐ |
| 1. When I think about dementia, I feel nauseous | ☐ | ☐ | ☐ | ☐ | ☐ |
| 1. The thought of dementia scares me | ☐ | ☐ | ☐ | ☐ | ☐ |
| 1. My feelings about myself would change if I developed dementia | ☐ | ☐ | ☐ | ☐ | ☐ |
| 1. There is a strong possibility that I will develop dementia | ☐ | ☐ | ☐ | ☐ | ☐ |
