# Supplementary material for: Public knowledge about dementia risk reduction in Norway
Source: BMC Public Health. 2022 Nov 8;22:2046. doi: 10.1186/s12889-022-14433-w (PMC9644554; doi:10.1186/s12889-022-14433-w)
Supplement: Supplementary file 3 — Additional file 3. Flowchart of the invited and participating sample. [file 12889_2022_14433_MOESM3_ESM.docx]

**Additional file 3. Flowchart of the invited and participating sample.**
